# Assessing inoculation’s effectiveness in motivating resistance to conspiracy propaganda in Finnish and United States samples

**DOI:** 10.3389/fpsyg.2024.1416722

**Published:** 2024-07-31

**Authors:** Elena Bessarabova, John A. Banas, Hanna Reinikainen, Neil Talbert, Vilma Luoma-aho, Katerina Tsetsura

**Affiliations:** ^1^Department of Communication, University of Oklahoma, Norman, OK, United States; ^2^Centre for Consumer Society Research, University of Helsinki, Helsinki, Finland; ^3^Center for Applied Social Research, University of Oklahoma, Norman, OK, United States; ^4^Jyväskylä University School of Business and Economics, University of Jyväskylä, Jyväskylä, Finland; ^5^Gaylord College of Journalism and Mass Communication, University of Oklahoma, Norman, OK, United States

**Keywords:** culture, inoculation, resistance, prebunking, motivational threat, thinking styles, conspiracies, 9/11 truth conspiracy

## Abstract

**Introduction:**

This study tested the motivational power of inoculation to foster resistance to conspiracy propaganda (*9/11 Truth* Movement), comparing inoculation effects across United States and Finnish study participants.

**Method:**

We used a 2 inoculation (treatment vs. control)  ×  2 national culture (American vs. Finnish) independent groups design (*N* = 319), while examining the effects of motivational threat and thinking modes—analytic vs. intuitive—on the inoculation process. To test the effectiveness of the inoculation strategy, we used an excerpt from a conspiracy film *Loose Change* as a counterattitudinal attack message.

**Results:**

Our results indicated that inoculation was effective at motivating resistance regardless of national culture. Inoculation effects emerged mostly as a direct effect on resistance and two indirect effects wherein motivational threat mediated the relationship between inoculation and resistance as well as inoculation and analytic mode of message processing. Although we found that an increase in analytic mode of processing facilitated resistance and intuitive processing increased conspiracy-theory endorsement, the indirect effects between inoculation and resistance via message processing modes were not significant. Finally, the data revealed national culture differences in analytic mode and cultural-context differences mostly pertaining to the relationships between thinking styles, media literacy, and modes of thinking.

**Discussion:**

These results offer important theoretical implications for inoculation scholarship and suggest viable practical solutions for efforts to mitigate misinformation and conspiratorial beliefs.

## 1 Introduction

Misinformation is a global problem. In the 2018 Global Risks Report, the World Economic Forum (2018) identified online misinformation as one of the most urgent global risks to the structures essential for our collective future, including environmental, economic, technological, and political systems. Conspiracy theories—causal narratives of events framed as covert malevolent plans orchestrated by a secret cabal as opposed to random or natural unfortunate events (Douglas and Sutton, 2008)—are a subtype of misinformation (Banas and Bessarabova, 2023). Often, the belief in misinformation is predicated on some sort of conspiracy theory. For example, the misinformed belief that climate change is a hoax assumes that scientists are collaborating throughout the world to deceive the public, making this a conspiracy theory (Cook, 2020). Misinformation and conspiratorial ideation should not be taken lightly because they have been shown to lead to a range of negative consequences, including reduced trust in democratic processes (Banas and Bessarabova, 2023), diminished scientific confidence regarding climate change (Cook et al., 2017; van der Linden et al., 2017), and doubts about the safety of vaccines (Kata, 2010; Banas et al., 2023; Bessarabova and Banas, 2024). For example, Andrew Wakefield’s anti-vaccination conspiracy propaganda, including his film *Vaxxed: From Cover-Up to Catastrophe,* is credited with increasing vaccine hesitancy and reducing childhood vaccinations across the globe (Deer, 2020). Similarly, the conspiracy theory that Bill Gates masterminded the COVID-19 pandemic and put microchips in the vaccine in order to control human behaviors were among the falsehoods that lowered vaccination intentions and compliance with guidelines issued by public health officials (Loomba et al., 2021; van der Linden, 2022).

The numerous problems caused by conspiracy theories and misinformation have led countries across the globe to consider countermeasures attempting to mitigate their influence. Finland, for instance, invested substantial federal funds to curb the spread of misinformation. The Finnish government created cross-sector initiatives, involving collaboration from all branches of government, academic experts, and the system of education, to better prepare citizens of all ages for the complexities of the modern-day digital landscape (Mackintosh, 2019). In Finland, media literacy starts in preschool and is taught as part of the core curriculum in all schools in the nation (Gross, 2023; National Audiovisual Institute, 2024). As a result of these efforts, Finland has been ranked first in Europe on misinformation resilience (Lessenski, 2022). Although the United States was not part of the misinformation resilience report, polls continue to show that the prevalence of misinformation in the United States is on the rise, with trust in official accounts at an all-time low (Gross, 2023). According to Gallup, in 2022, only 7% of respondents fully trusted traditional media sources to report information accurately and fairly (Brenan, 2022). In comparison, 76% of Finns hold print and digital news media in high regard (Newman et al., 2023). Further, in the United States school curriculum, media literacy is more likely to be taught as a class or special seminar geared toward older students instead of being part of the national curriculum taught to students of all ages (Center for an Informed Public, 2023). Despite these differences, the misinformation problems that both countries face are remarkably similar. According to a report on the disinformation landscape in Finland, online trolls, trust in alternative media, election disinformation, COVID-19 conspiracies, and nationalist propaganda are important concerns that Finns encounter in their ongoing information warfare (Moilanen et al., 2023). Very similar problems also plague the United States. Thus, examining motivational strategies that might be effective at misinformation and conspiracy-theory prevention across different cultural groups has considerable theoretical and practical importance.

One such motivational strategy that has been shown to be effective at preventing the spread of misinformation and conspiracy-theory endorsement is based on McGuire’s (1961) theory of inoculation. We seek to advance inoculation and conspiracy theory misinformation research in three notable ways. First, this study focuses on the effects of one of the key mechanisms of inoculation, motivational threat (Banas and Richards, 2017). Although scholarship examining inoculating against misinformation has been prolific recently, most research ignores the mechanisms that account for inoculation effects (e.g., Roozenbeek et al., 2020; Lees et al., 2023). Second, the present study examines how inoculation affects modes of thinking and how those thinking modes are related to resistance. Analytic thinking has been shown to be negatively related, and intuitive thinking has been shown to be positively related, to conspiracy theory misinformation endorsement (Swami et al., 2014), but research is less clear on how message interventions affect thinking modes, especially in the context of inoculating against conspiracy-theory misinformation. Third, this study examines cultural differences in the process of inoculating against conspiracy-theory misinformation. Cross-cultural validations of inoculation with non-United States samples have been mixed (e.g., Ivanov et al., 2012; Roozenbeek et al., 2020; Spampatti et al., 2024), and comparing United States and Finnish samples can lend insights into the robustness of inoculation effects as well as nuances in transporting inoculation treatments to other countries.

Inoculation theory utilizes medical inoculation as a metaphor for inducing resistance to persuasion. Similar to how conventional biological inoculations work by introducing people to a weakened form of a virus to induce the production of antibodies to resist infection, McGuire (1961) believed that exposing people to weakened persuasive arguments would stimulate attitudinal defenses. According to inoculation theory, vulnerability to counterattitudinal persuasion is greatest when people are unmotivated and unpracticed at defending their beliefs.

Modern inoculation message treatments typically contain two main message features: explicit forewarning and refutational preemption, which are designed to trigger the theoretical mechanisms of perceived threat and counterarguing (Pfau et al., 1997; Parker et al., 2012; Wong, 2016). The forewarning is designed to highlight one’s attitudinal vulnerability to potential impending counterpersuasion, thus motivating a range of resistance-promoting actions. The refutational preemption typically involves using two-sided messages that raise and then refute arguments (Compton, 2013).

Inoculation interventions have been effective in a variety of different contexts (see Banas and Rains, 2010, for a meta-analysis), and research on inoculation has increasingly been applied to misinformation generally (e.g., Cook et al., 2017; Roozenbeek et al., 2020; Lees et al., 2023) and conspiracy-theory endorsement specifically (e.g., Banas and Miller, 2013; Banas et al., 2023; Bessarabova and Banas, 2024). Misinformation inoculation research has been driven by brilliant and inventive gamification interventions, such as *Bad News*. Through playing games where participants actively produce misinformation (e.g., Roozenbeek and van der Linden, 2019) or promote division (Roozenbeek and van der Linden, 2020), dynamic and sophisticated inoculation interventions have been shown to reduce the credibility of misinformation. In contrast, conspiracy theory inoculation research has been more dynamic in terms of the attack messages being used than in the inoculation treatments. In studies inoculating against *9/11 Truth* propaganda, conventional page-long essays inoculate against a 40-min clip from the conspiracy theory film, *Loose Change: Final Cut* (Banas and Miller, 2013; Banas and Richards, 2017). Similarly, Banas et al. (2023) used a page-long essay to effectively inoculate against a 40-min clip from the anti-vaccination conspiracy theory movie, *Vaxxed: From Cover-up to Catastrophe*. Importantly, regardless of the type of inoculation treatment or counterattitudinal attack message, inoculation (with a few exceptions; see Spampatti et al., 2024) appears to be an effective motivational strategy for resistance production. Based on the premises of inoculation theory, we predict:

*H1*: Relative to the control condition, inoculation reduces positive attitudes toward conspiracy propaganda (i.e., facilitates resistance to conspiracy propaganda).

A relatively recent development in inoculation research is the introduction of motivational threat (Banas and Richards, 2017) as an important mechanism of resistance. Inspired by the meta-analytic finding that perceived threat was not significantly correlated with resistance (Banas and Rains, 2010), Banas and Richards (2017) designed a new measure of the threat mechanism of inoculation that focused more on the *motivation* to engage in attitudinal defenses and less on the *apprehensive state* captured in the traditional measure. The Banas and Richards’ (2017) measure has been subsequently validated, with studies demonstrating that motivational threat is positively associated with resistance (e.g., Richards and Banas, 2018; Ivanov et al., 2020). However, there are currently no studies of motivational threat with non-United States participants.

Motivational threat is likely to increase attentional focus on the information presented in the inoculation message and prompt more deliberate, analytic message processing as a result. Deliberate engagement with the inoculation material makes sense, given that in inoculation research (e.g., Pfau et al., 2003, 2004) the refutational preemption is based on logic and grounded in statistical evidence and verifiable research that should trigger analytic processing and reduce reliance on intuition and gut reactions. Previous studies on inoculation have not examined the relationship between motivational threat and different modes of cognitive processing (i.e., analytic vs. intuitive), which is a novel contribution of this research. Based on this rationale, we predict:

*H2*: (a) Relative to the control condition, inoculation increases motivational threat, which subsequently (b) increases analytic mode of thinking and (c) decreases intuitive mode of message processing.

An increase in analytic thinking should facilitate resistance to persuasion, whereas intuitive message processing should be counterproductive and consequently dampen resistance to conspiracy propaganda. Consistent with this reasoning, Swami et al. (2014) found intuitive thinking to be positively related, and analytic thinking to be negatively related, to conspiracy-theory endorsement (Study 1). In addition to these correlational findings, Swami et al. (2014) showed that priming analytic thinking reduced belief in an inventory of conspiracy theories (measuring people’s endorsement of the New World Order conspiracy theory among others), relative to the control condition (Studies 2–4). The notion that promoting a more analytic mode of message processing can be effective at reducing conspiracy-theory belief is also supported by the work of Adam-Troian et al. (2019), who found that priming rationality enhanced the negative relationship between cognitive ability and conspiracy-theory endorsement. In a systematic review of interventions designed to reduce conspiratorial ideation, O’Mahony et al. (2023) concluded that two types of interventions were most effective: inoculation interventions and interventions that promoted analytic or critical thinking. As our study features an inoculation treatment that, among other things, seeks to promote an analytic message processing mode, we predict:

*H3*: Analytic mode of thinking reduces, and intuitive message processing increases, positive attitudes toward conspiracy propaganda.

Despite the overwhelming support for inoculation’s effectiveness, the results of cross-cultural validations of inoculation have been mixed. For instance, Roozenbeek et al. (2020) tested the effectiveness of their *Bad News* videogame—informing players about several common misinformation techniques—in Sweden, Germany, Greece, and Poland, and found that videogame strategies based on inoculation were effective at facilitating resistance to online misinformation in all countries they sampled. Similarly, Ivanov et al. (2012) examined inoculation effects comparing United States college students (a low-context, individualistic culture) to Asian-American and East-Asian college participants (a high-context, collectivistic culture), finding that inoculation can protect attitudes regardless of whether inoculations were tailored to a particular culture. However, in a recent study, Spampatti et al. (2024) recruited participants from the United States, Canada, the United Kingdom, Ireland, Australia, New Zealand, Singapore, the Philippines, India, Pakistan, Nigeria, and South Africa to test inoculation’s effectiveness against climate change denial and found “almost no evidence for protective effects of the inoculations” (p. 380). In light of these mixed results, we ask:

*RQ1*: Does national culture affect resistance?

Cultural validations like Ivanov et al. (2012), Roozenbeek et al. (2020), and Spampatti et al. (2024) are rare and important for research on inoculation, yet neither of these studies tried to examine whether there are cultural influences beyond national culture affecting inoculation. Theoretically, there is good reason to focus on specific dimensions of cultural differences rather than examining cultures at the national level. Culture at the national level is too broad a construct to be meaningful as an explanatory variable: A more nuanced approach to examine differences across societal groups is to focus on the specific cultural factors, called *cultural-context variables* (Poortinga and van de Vijver, 1987; Bond and van de Vijver, 2010; van de Vijver and Leung, 2021). In cross-cultural research, context variables, such as self-construals (Markus and Kitayama, 1991) or Hofstede’s (1984) dimensions, can provide interpretable explanations that account for differences in a variety of outcome variables (e.g., Oyserman and Lee, 2008; Ma-Kellams and Blascovich, 2012; Park et al., 2012; Yuki and Schug, 2020; Ma et al., 2021; Machette and Cionea, 2022). Considering cultural differences related to information processing and conspiracy endorsement, we focused on individual differences in the cultural-context variables of (1) thinking styles (rational vs. experiential), (2) differences in media literacy, and (3) epistemic trust.

First, grounded in cognitive-experiential self-theory (CEST; Epstein, 1994), *the rational thinking style* is defined as “an inferential system that operates by a person’s understanding of culturally transmitted rules of reasoning; it is conscious, relatively slow, analytical, primarily verbal, and relatively affect-free; and it has a very brief evolutionary history,” whereas *the experiential thinking style* is conceptualized as “a learning system that is preconscious, rapid, automatic, holistic, primarily nonverbal, intimately associated with affect, and it has a very long evolutionary history” (Pacini and Epstein, 1999, p. 972). Extant research demonstrates that experiential thinking style increases beliefs that are at odds with scientific explanations, including paranormal beliefs (Aarnio and Lindeman, 2005; Lobato et al., 2014; Majima et al., 2022), beliefs in the supernatural (Gervais and Norenzayan, 2012; Pennycook et al., 2012, 2014), and pseudoscientific beliefs (Lindeman, 2011). For example, when Shenhav et al.’s (2012) participants were tasked with the cognitive reflection test (Frederick, 2005) involving a series of easy-to-solve math problems that had “intuitively compelling incorrect answers,” selecting the more intuitive responses to the task was positively associated with stronger endorsement of supernatural beliefs (Gervais and Norenzayan, 2012; p. 423). Similarly, Swami et al. (2014) found that conspiratorial beliefs were positively associated with intuitive thinking and negatively associated with analytic thinking. Thus, cultural differences in thinking styles might influence the mechanisms of inoculation.

Second, the differences in media literacy discussed above—state-level educational interventions to combat misinformation in Finland, and, comparatively, more modest attempts to prevent misinformation in the United States (Center for an Informed Public, 2023)—are also likely to influence the mechanisms of inoculation. As research indicates, participating in media-literacy programs has positive motivational influences on information-seeking intentions, knowledge of media, and the ability to analyze the news (Martens and Hobbs, 2015). Furthermore, meta-analytic findings reveal media literacy decreases conspiracy-theory endorsement (Jeong et al., 2012). As concluded by Craft et al. (2017), the “greater one’s knowledge about the news media—from the kinds of news covered, to the commercial context in which news is produced, to the effects on public opinion news can have—the less likely one will fall prey to conspiracy theories” (p. 400). Overall, it is reasonable to expect cultural differences in media literacy to have an effect on the mechanisms of inoculation.

The third cultural-context variable we focused on is epistemic trust. The socio-epistemic model of belief in conspiracy theories states that epistemic mistrust is the fundamental driver of all conspiracy-theory belief (Pierre, 2020). Once authorities and official institutions are not considered credible, the epistemic vacuum can lead people to engage in biased message processing and become vulnerable to believing misinformation that supports conspiracy-theory beliefs, especially in the current media and social-media ecosystems, which are populated with false news. As discussed, the United States and Finland are quite different with regard to trust in epistemic authorities (Center for an Informed Public, 2023), thus, we expect epistemic trust to influence the effects of inoculation treatments attempting to prevent conspiratorial influences.

Given that the effects of cultural-context variables—thinking styles (rational vs. experiential), media literacy, and epistemic trust—have not been established in the context of inoculation research generally, as well as the differences in the United States vs. Finnish populations on these context variables as they relate to inoculation specifically, we pose the following research question:

*RQ2*: How do cultural-context variables—thinking styles, media literacy, and epistemic trust—affect mechanisms of resistance?

## 2 Materials and methods

### 2.1 Participants

A sample of 319 participants was recruited at two locations.^1^ To estimate whether the sample size was adequate to test hypotheses in the study, we used G*Power (Faul et al., 2007) to determine the sample size necessary for MANCOVA and Schoemann et al.’s (2017) software to estimate power for indirect effects in serial mediations. For G*Power analysis, we used a small/medium effect (*f*^2^ = 0.04), power set at 0.80, and 95% confidence interval. The results indicated that a sample size of 289 was sufficient to detect significant effects. For the mediation analysis, we conducted power estimation for a mediation model with two serial mediators and assumed small/medium effects by setting standardized coefficients to 0.20. Note that setting standardized path coefficients to 0.20 is reasonable based on previous inoculation research (see Banas and Rains’, 2010, meta-analysis). The results of the power analysis indicated that for power set at 0.80 and 95% confidence interval, a sample of 310 participants was required. Based on these results, our sample size of 319 participants was adequate to detect significant effects.

The United States sample (*n* = 171) was obtained from a research participation pool at a large public university in the South-Central part of the United States. The Finnish sample (*n* = 148) came from a large university in the Central part of Finland. In the study, 57.7% of participants self-identified as females. The majority (56%) were Freshmen (32%) and Sophomores (24%). Consistent with the EU guidelines protecting research participants’ personal information, no direct identifiers were collected. To further protect participants’ identities, their age was measured in ranges (e.g., 18–19, 20–21). The majority of participants (66.8%) were regular college-age: 18–22 years of age, with an age range between 18 and 29 years of age.

### 2.2 Design and procedure

To test study predictions, a 2 inoculation (treatment vs. control) × 2 national culture (American vs. Finnish) independent groups design was employed. The data at both locations were collected in person. As with previous studies on inoculation (e.g., Banas and Miller, 2013), the data collection comprised three phases. In Phase 1, participants completed informed consent procedures and were asked to respond to measures of cultural differences: (1) thinking styles (rational and experiential), (2) epistemic trust, and (3) media literacy. Participants then reported their socio-demographic characteristics and responded to questionnaire items measuring their initial endorsement of the conspiracy that the United States government perpetrated the attacks on 9/11. In Phase 2, which immediately followed Phase 1, participants were randomly assigned to one of the experimental conditions: inoculation treatment or control (see the description below). Next, participants answered motivational threat items, followed by the mode of information processing questions. In Phase 3, all participants were exposed to a counterattitudinal attack—a 30-min excerpt from the film *Loose Change*—explicitly making the case for the conspiracy that the United States government orchestrated 9/11. After watching the film, participants’ responded to questions measuring their attitudes toward the conspiracy film. The study concluded with questions measuring the self-reported ease of comprehension. All participants were then thoroughly debriefed.

### 2.3 Inoculation materials

The experimental materials—the inoculation treatment and control message—came from Banas and Miller (2013). Consistent with previous research (Banas and Rains, 2010), the inoculation treatment comprised a forewarning and a refutational preemption. Forewarning was manipulated by alerting participants that they might come across a compelling conspiracy film causing them to rethink what they know about the 9/11 attacks on American soil. The inoculation treatment contained a series of arguments (i.e., refutations) systematically disputing the logic and facts of the 9/11 conspiracy theory. Neither the forewarning nor the refutational preemption were part of the control condition. Instead, the control condition presented a page-long description of the history of sushi, containing identical wording from Banas and Miller (2013).

### 2.4 Instrumentation

All variables were transformed to help meet the assumptions of the general linear model (Fink, 2009). All indices were formed using a principal component analysis (PCA) with an unrotated one-component solution; as a result of this procedure, standardized regression component scores were saved for each participant (Afifi et al., 2004).^2^ Indices formed employing this commonly used approach have *M* = 0.00, *SD* = 1 with range ≈ −3 to +3; in this method, each index item is weighted based on its contribution to the principal component. The following formula was used to compute reliabilities: *N*/(*N*-1) × (*E*-1)/*E*, wherein *N* = the number of items and *E* = Eigenvalues for principal components (Serlin and Kaiser, 1976; Hampson et al., 1987). PCA reliabilities are reported throughout.

#### 2.4.1 Thinking styles

The rational-experiential inventory was used to capture rational and experiential thinking styles (Pacini and Epstein, 1999). A separate index for each thinking style was created. The rational thinking style scale (e.g., “I enjoy thinking in abstract terms;” PCA reliability = 0.89) and intuitive thinking style scale (e.g., “I like to rely on my intuitive impressions;” PCA reliability = 0.91) each comprised 20 items. A 9-point Likert-type scale was used, wherein 1 = *strongly disagree* and 9 = *strongly agree*.

#### 2.4.2 Epistemic trust

Participants were asked to what extent they trusted different segments of the society—religious, educational institutions, as well as interpersonal connections such as friends, family, and neighbors—to do the right thing, using Mmari et al.’s (2016) community trust scale. A 9-point Likert-type scale was used, wherein 1 = *no trust at all* and 9 = *a lot of trust*. PCA reliability = 0.68.

#### 2.4.3 Media literacy

Six items (e.g., “I think about … the purpose behind a message I see in the media; … who created a message I see in the media; … what the people who made a media message want me to believe; … what a media message did to get my attention;” Scull et al., 2010) were used to capture the extent to which participants think critically about media messages. A 9-item scale was used, wherein 1 = *never* and 9 = *always*. PCA reliability = 0.83.

#### 2.4.4 Motivational threat

We used Banas and Richards’ (2017) measure to capture motivational threat, assessed immediately after the inoculation induction. The scale comprised 4 Likert-type items (e.g., “I want to defend my current attitudes from attack”) measured on a 9-point Likert-type scale, wherein 1 = *strongly disagree* and 9 = *strongly agree*. PCA reliability = 0.80.

#### 2.4.5 Initial attitudes and resistance

Burgoon et al.’s (1978) attitude measure (e.g., negative/positive, unfavorable/favorable, wrong/right) was used to capture participants’ initial attitudes toward the idea that the United States government participated in a conspiracy to perpetrate the attacks on 9/11 (measured at Phase 1; PCA reliability = 0.88) and attitudes toward the position advocated in the conspiracy film (i.e., an excerpt from *Loose Change* arguing that 9/11 was an inside job, captured at Phase 3; PCA reliability = 0.93); the latter measure was used to ascertain resistance. A standard approach in inoculation research is to measure resistance by comparing attitudes—here, to conspiracy propaganda—of inoculated participants relative to control. If attitudes in the inoculation condition are less positive than in the control, resistance is inferred (Banas and Rains, 2010). To measure attitudes, a 1 to 9 scale was used. Consistent with previous studies on inoculation (e.g., Ivanov et al., 2009; Pfau et al., 2009, 2010), we covaried initial attitudes to be able to better capture the unique influences of the inoculation treatment.

#### 2.4.6 Issue involvement

Defined in terms of issue importance, involvement was measured with an abbreviated personal involvement inventory (Zaichkowsky, 1985). A 5-item Likert-type scale was used (insignificant/significant, unimportant/important, of no concern/of much concern, means nothing/means a lot, irrelevant/relevant, and does not/does matter to me; PCA reliability = 0.93), ranging from 1 to 9. In line with previous research (e.g., Ivanov et al., 2009; Pfau et al., 2009, 2010), we covaried issue involvement to better determine the unique effects of our inoculation strategy on resistance.

#### 2.4.7 Message processing mode: analytic vs. intuitive

We used Novak and Hoffman’s (2009) measure to capture message processing mode. To measure the analytic mode of processing, an 8-item subscale was used (e.g., “I reasoned things out carefully;” PCA reliability = 0.91); 10 items were used to assess the intuitive mode of processing (e.g., “I relied on my first impressions;” “I used my gut feelings;” PCA reliability = 0.93). A 9-point Likert-type scale, wherein 1 = *definitely false* and 9 = *definitely true*, was employed to measure different modes of message processing.

#### 2.4.8 Ease of comprehension

We asked study participants to self-report how easy it was to understand the study information (*M*_Fin_ = 6.29, *SD*_Fin_ = 1.80; *M*_U.S._ = 7.32, *SD*_U.S._ = 1.69), survey questions (*M*_Fin_ = 5.82, *SD*_Fin_ = 1.90; *M*_U.S._ = 7.15, *SD*_U.S._ = 1.79), and the video they watched (*M*_Fin_ = 6.95, *SD*_Fin_ = 1.67; *M*_U.S._ = 7.84, *SD*_U.S._ = 1.35). The means for comprehension across both samples were above the midpoint, although the United States participants’ ratings were slightly higher, relative to Finns. Finns, however, are very proficient in English, given that 85% of the Finnish population learns English starting as early as primary school (Devlin, 2020). Thus, their English proficiency was sufficient to participate in this research. Given the above descriptives, we formed a comprehension index and covaried its effects in the analyses that follow.

## 3 Results

For bivariate correlations between all variables in the study, see Table 1.

**Table 1:** Bivariate correlations between all variables in the study.

	1	2	3	4	5	6	7	8	9	10	11	12	13
1. National culture	1.00												
2. Inoculation	0.05	1.00											
3. Motivational threat	0.19^**^	0.20^**^	1.00										
4. Analytic mode	0.30^**^	0.29^**^	0.41^**^	1.00									
5. Intuitive mode	0.14^*^	0.04	0.17^**^	0.12^*^	1.00								
6. Consp. film attitude	−0.03	−0.21^**^	−0.25^**^	−0.22^**^	0.07	1.00							
7. Rational trait	−0.05	0.07	0.15^**^	0.35^**^	−0.21^**^	−0.16^**^	1.00						
8. Experiential trait	0.16^**^	0.05	0.09	0.08	0.49^**^	0.01	−0.11^*^	1.00					
9. Epistemic trust	0.05	0.10	0.24^**^	0.16^**^	0.16^**^	−0.02	0.07	0.10	1.00				
10. Media literacy	0.05	0.05	0.14^*^	0.32^**^	0.02	−0.07	0.25^**^	0.01	0.01	1.00			
11. Initial attitudes	−0.18^**^	0.02	−0.26^**^	−0.16^**^	−0.13^*^	0.26^**^	−0.02	−0.15^**^	−0.12^*^	−0.04	1.00		
12. Issue involvement	0.48^**^	0.04	0.38^**^	0.25^**^	0.23^**^	−0.01	−0.07	0.19^**^	0.23^**^	0.09	−0.41^**^	1.00	
13. Comprehension	0.39^**^	0.11^*^	0.37^**^	0.46^**^	0.07	−0.08	0.21^**^	0.10	0.19^**^	0.25^**^	−0.14^*^	0.33^**^	1.00

*p < 0.05; ^**^p < 0.01. Consp. film attitude stands for conspiracy film attitude (i.e., resistance). For national culture, Finnish sample = 1, the United States sample = 2.

### 3.1 Analytic strategy

The cultural effects, RQ1–2, were examined in a multivariate analysis of covariance (MANCOVA), and the mechanisms of inoculation, H1–H4, were tested in PROCESS v.4 (Hayes, 2022).

### 3.2 Hypotheses tests and examination of research questions

#### 3.2.1 RQs 1 and 2: the effects of national culture and context variables

In the MANCOVA, inoculation treatment and national culture were entered as independent variables; rational and experiential thinking styles, epistemic trust, media literacy, attitudes toward the idea that the United States government participated in a conspiracy to perpetrate the attacks on 9/11 measured at Phase 1 (i.e., initial attitudes), issue involvement measured at Phase 1, and ease of comprehension were entered as covariates; motivational threat, analytic message processing mode, intuitive message processing mode, and attitudes toward the position advocated in the conspiracy film (i.e., resistance) measured at Phase 3 were entered as dependent variables. The multivariate effect of inoculation treatment, Wilk’s Λ = 0.87, *F*(4, 277) = 10.38, *p* < 0.001, η_p_^2^ = 0.13, was significant, and the multivariate effect of national culture, Wilk’s Λ = 0.97, *F*(4, 277) = 2.30, *p* = 0.059, η_p_^2^ = 0.03, and the interaction between national culture and inoculation treatment, Wilk’s Λ = 0.98, *F*(4, 277) = 1.22, *p* = 0.303, η_p_^2^ = 0.02, were not significant. The multivariate effects of covariates—rational thinking style, Wilk’s Λ = 0.87, *F*(4, 277) = 10.64, *p* < 0.001, η_p_^2^ = 0.13, experiential thinking style, Wilk’s Λ = 0.80, *F*(4, 277) = 17.58, *p* < 0.001, η_p_^2^ = 0.20, media literacy, Wilk’s Λ = 0.96, *F*(4, 277) = 2.71, *p* = 0.031, η_p_^2^ = 0.04, initial attitudes toward the notion that the United States government perpetrated 9/11, Wilk’s Λ = 0.92, *F*(4, 277) = 6.36, *p* < 0.001, η_p_^2^ = 0.08, issue importance, Wilk’s Λ = 0.92, *F*(4, 277) = 5.72, *p* < 0.001, η_p_^2^ = 0.08, and ease of comprehension, Wilk’s Λ = 0.93, *F*(4, 277) = 5.56, *p* < 0.001, η_p_^2^ = 0.07—were significant, whereas the effect of epistemic trust was not significant, Wilk’s Λ = 0.97, *F*(4, 277) = 2.00, *p* = 0.095, η_p_^2^ = 0.03.

The univariate effects are summarized in Table 2. In response to RQ1, asking whether national culture affects resistance, we found limited influences of national culture on the mechanisms of inoculation: Only analytic processing mode was affected by national culture. Specifically, United States participants (*M* = 0.29, *SD* = 0.97) were significantly more likely to use analytic mode of processing, *F*(1, 289) = 7.03, *p* = 0.008, η_p_^2^ = 0.02, relative to Finnish participants (*M* = −0.29, *SD* = 0.95). For this effect, rational thinking style, media literacy, and ease of comprehension emerged as significant covariates. All other effects of national culture or its interaction with inoculation treatment were not significant. Thus, it appears that national culture only affected one mechanism of inoculation—analytic processing mode—and did not have an effect on either motivational threat, intuitive mode of processing, or resistance.

**Table 2:** MANCOVA results.

Covariates/independent variables	Dependent variables	*F*	*p*	η_p_^2^
Rational trait	Motivational threat	2.19	0.140	0.01
	Analytic mode	28.14	<0.001	0.09
	Intuitive mode	8.92	0.003	0.03
	Consp. film attitude (resistance)	4.04	0.045	0.01
Experiential trait	Motivational threat	0.03	0.854	0.00
	Analytic mode	0.74	0.391	0.00
	Intuitive mode	69.79	<0.001	0.20
	Consp. film attitude (resistance)	0.10	0.755	0.00
Epistemic trust	Motivational threat	3.64	0.057	0.01
	Analytic mode	0.60	0.438	0.00
	Intuitive mode	5.01	0.026	0.02
	Consp. film attitude (resistance)	0.04	0.838	0.00
Media literacy	Motivational threat	0.27	0.605	0.00
	Analytic mode	10.87	0.001	0.04
	Intuitive mode	0.33	0.567	0.00
	Consp. film attitude (resistance)	0.10	0.748	0.00
Initial attitudes	Motivational threat	4.71	0.031	0.02
	Analytic mode	2.61	0.107	0.01
	Intuitive mode	0.02	0.875	0.00
	Consp. film attitude (resistance)	23.45	<0.001	0.08
Issue involvement	Motivational threat	15.43	<0.001	0.05
	Analytic mode	2.14	0.144	0.01
	Intuitive mode	2.58	0.109	0.01
	Consp. film attitude (resistance)	2.72	0.100	0.01
Comprehension	Motivational threat	11.44	<0.001	0.04
	Analytic mode	12.78	<0.001	0.04
	Intuitive mode	0.22	0.637	0.00
	Consp. film attitude (resistance)	0.01	0.937	0.00
National culture	Motivational threat	0.78	0.378	0.00
	Analytic mode	7.03	0.008	0.02
	Intuitive mode	0.14	0.711	0.00
	Consp. film attitude (resistance)	0.08	0.777	0.00
Inoculation	Motivational threat	7.65	0.006	0.03
	Analytic mode	29.21	<0.001	0.09
	Intuitive mode	0.02	0.892	0.00
	Consp. film attitude (resistance)	15.62	<0.001	0.05
National culture × Inoculation	Motivational threat	0.09	0.762	0.00
	Analytic mode	0.05	0.817	0.00
	Intuitive mode	3.10	0.079	0.01
	Consp. film attitude (resistance)	2.06	0.152	0.01

Consp. film attitude = Conspiracy film attitude; for all analyses, df = 1, 280.

RQ2 inquired how cultural-context variables—thinking styles, media literacy, and epistemic trust—affect mechanisms of resistance. As evident from Table 2, rational thinking style covaried with analytic mode of processing, intuitive mode of processing, and attitudes toward conspiracy film measured at Phase 3 (i.e., resistance). The experiential thinking style covaried only with intuitive mode of processing. Finally, media literacy covaried with analytic mode of processing. The fact that different thinking styles and media literacy covaried with the two modes of thinking—analytic and intuitive—is not surprising: Given the nature of questions measuring these constructs, they are bound to covary. Thus, with the exception of rational thinking style, neither of the cultural-context measures had significant influences on the core mechanisms of inoculation: motivational threat and resistance.

#### 3.2.2 Hypotheses 1–4: mechanisms of inoculation

**Figure 1:**
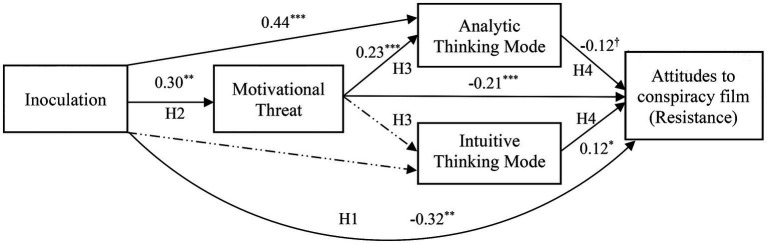
Mediation model 81 with unstandardized coefficients. ^†^*p* = 0.031 (one-tailed); ^*^p < 0.05 (two-tailed); ^**^p < 0.01 (two-tailed); ^***^p < 0.001 (two-tailed). Dashed lines represent nonsignificant relationships. The solution involves initial attitudes toward conspiracy, issue involvement, and ease of comprehension used as covariates.

To examine mechanisms of inoculation, we conducted a mediation analysis in PROCESS v.4 (Hayes, 2022, Model 81). In the mediation model, inoculation treatment was entered as the independent variable; motivational threat as a mediator, followed by analytic mode of message processing and intuitive mode of message processing as two subsequent parallel mediators; to ascertain resistance, positive attitudes toward the position advocated in the film were entered as the dependent variable. We used national culture, initial attitudes, and issue involvement as covariates. We did not enter cultural-context variables as covariates into the mediation model. Several factors influenced this decision. PROCESS does not allow entering custom covariates for each consequent (i.e., mediators and the dependent variable) in the model. Thus, if we were to enter cultural-context variables into the mediation analysis, the same set of covariates would be used in each regression performed for each consequent. Given that, based on the MANCOVA results, the significant influences of cultural-context variables were found mostly for the mode of processing variables, using cultural-context variables as covariates across the board did not seem reasonable. As evident from the bivariate correlations between all variables in the study (see Table 1), a small yet significant correlation between inoculation and ease of comprehension was found, indicating that the control message was slightly easier to understand, relative to the inoculation message. Thus, in addition to national culture, initial attitudes, and issue involvement, we covaried the effects of ease of comprehension in our mediation analysis. The summary of mediation results is presented in Table 3; for graphic representation, see Figure 1.

**Table 3:** Mediation model 81 results with unstandardized coefficients.

Consequent												
	*M_1_* (Motivational Threat)			*M_2_* (Analytic Mode)			*M_3_* (Intuitive Mode)			*Y* (Consp. Film Att., i.e., Resistance)		
Antecedent	Coeff.	*SE*	*p*	Coeff.	*SE*	*p*	Coeff.	*SE*	*p*	Coeff.	*SE*	*p*
*X* (Inoculation)	0.30	0.10	0.004	0.44	0.10	<0.001	0.02	0.12	0.840	−0.32	0.11	0.006
*M_1_* (Motivational Threat)	–	–	–	0.23	0.05	<0.001	0.08	0.06	0.214	−0.21	0.06	0.001
*M_2_* (Analytic Mode)	–	–	–	–	–	–	–	–	–	−0.12	0.07	0.062
*M_3_* (Intuitive Mode)	–	–	–	–	–	–	–	–	–	0.12	0.06	0.031
*C_1_* (National Culture)	−0.16	0.12	0.179	0.22	0.11	0.056	0.15	0.13	0.270	−0.06	0.13	0.641
*C_2_* (Initial Attitudes)	−0.14	0.06	0.020	−0.06	0.06	0.260	−0.04	0.07	0.602	0.29	0.07	< 0.001
*C_3_* (Issue Involvement)	0.27	0.06	<0.001	0.02	0.06	0.729	0.17	0.07	0.019	0.19	0.07	0.007
*C_4_* (Comprehension)	0.27	0.06	<0.001	0.26	0.05	<0.001	−0.06	0.06	0.327	0.06	0.06	0.368
Constant	0.11	0.20	0.594	−0.56	0.19	0.003	−0.22	0.22	0.321	0.24	0.22	0.271
	*R*^2^ = 0.24, *F*(5, 291) = 18.87, *p* < 0.001			*R*^2^ = 0.32, *F*(6, 290) = 23.13, *p* < 0.001			*R*^2^ = 0.06, *F*(6, 290) = 3.30, *p* = 0.004			*R*^2^ = 0.18, *F*(8, 288) = 7.95, *p* < 0.001		

Conspir. Film Att. = Conspiracy Film Attitudes (i.e., resistance); Comprehension = Ease of Comprehension.

**Table 4:** Mediation model 4 with unstandardized coefficients to estimate the indirect effect for inoculation➔motivational threat➔analytic mode relationship.

	Consequent					
	*M_1_* (Motivational Threat)			*M_2_* (Analytic Mode)		
Antecedent	Coeff.	*SE*	*p*	Coeff.	*SE*	*p*
*X* (Inoculation)	0.32	0.10	0.002	0.41	0.10	<0.001
*M_1_* (Motivational Threat)	–	–	–	0.23	0.05	<0.001
*C_1_* (National Culture)	−0.16	0.12	0.196	0.23	0.11	0.050
*C_2_* (Initial Attitudes)	−0.15	0.06	0.014	−0.05	0.06	0.343
*C_3_* (Issue Involvement)	0.27	0.06	<0.001	0.00	0.06	0.961
*C_4_* (Ease of Comprehension)	0.27	0.06	<0.001	0.29	0.05	<0.001
Constant	0.08	0.20	0.677	−0.56	0.19	0.003
	*R*^2^ = 0.25, *F* (5, 298) = 20.02, *p* < 0.001			*R*^2^ = 0.32, *F* (6, 297) = 23.45, *p* < 0.001		

Consistent with H1, we found inoculation had a significant direct effect on resistance. As hypothesized in H2a, inoculation increased motivational threat, and, in support of H2b, motivational threat increased analytic mode of thinking. However, the effect between motivational threat and intuitive thinking mode was not significant, contrary to H2c. Finally, consistent with H3, analytic thinking mode increased resistance, whereas intuitive thinking mode was positively associated with the positive attitudes toward the conspiracy film. In the model, a significant mediation (indirect effect = −0.06, BootSE = 0.03, BootCI [−0.13, −0.01]; direct effect = −0.32, BootSE = 0.11, BootCI [−0.55, −0.10]) was found for the relationship between inoculation and resistance via motivational threat. Another significant indirect effect emerged for the relationship between inoculation and analytic thinking mode via motivational threat (indirect effect = 0.07, BootSE = 0.03, BootCI [0.02, 0.15]; direct effect = 0.41, BootSE = 0.10, BootCI [0.22, 0.61]), indicating that the inoculation treatment increased motivational threat, which subsequently increased analytic mode of processing.^3^ All other indirect effects were not significant.^4^

## 4 Discussion

Conspiracy theories and misinformation continue to pose a serious threat to societies across the world, and our study contributes to a growing literature on how inoculation theory can be effectively applied to help prevent their spread. In our study, a brief inoculation treatment in the form of a one-page essay induced resistance to a 30-min clip from a conspiracy theory propaganda film, and the effects were cross-culturally validated, as the process was identical for both American and Finnish participants. Our results are consistent with Ivanov et al.’s (2012) findings of inoculation’s effectiveness comparing the United States participants to Asian-American and East-Asian participants as well as the cross-cultural validation of Roozenbeek et al.’s (2020) *Bad News* videogame, whose inoculation intervention emerged as an effective inhibitor of misinformation credibility in samples from Sweden, Germany, Greece, and Poland. Although the *Bad News* intervention is quite different from the conventional message used in the present study, both that study and this one used European samples.

One of the main foci of this study was to examine how motivational threat affects the process of inducing resistance to conspiracy-theory misinformation. Although there has been prior empirical support for motivational threat as a key mechanism of the inoculation process (Banas and Richards, 2017; Richards and Banas, 2018; Ivanov et al., 2020), a recent study about inoculating against anti-vaccination conspiracy theory misinformation (Banas et al., 2023) found mixed results for motivational threat as a mediator. Our study provides support for motivational threat as a central mechanism of inoculation effects since motivational threat was a significant mediator of the relationship between inoculation and resistance as well as inoculation and analytic message processing.

The focus on message processing states, and how they are activated by motivational threat, is understudied in inoculation research. This is unfortunate because analytic thinking has been shown to be negatively related, and intuitive thinking has been shown to be positively related, to conspiracy-theory endorsement (e.g., Swami et al., 2014). As such, examining the role of message processing states has the potential to inform the process of inoculating against conspiracy-theory endorsement. Our study found that motivational threat increased analytic message processing states but was not related to intuitive processing states; however, regardless of inoculation condition, intuitive processing states were positively related to the endorsement of conspiracy-theory propaganda. Future scholarship should continue to examine the ways in which inoculation interventions can activate analytic message processing states to combat conspiracy-theory endorsement.

Related to the discussion of message processing states, we also would like to acknowledge that analytic and intuitive modes of processing in our study were positively correlated. When building the rationale for the relationships between motivational threat and message processing modes, we hypothesized these relationships based on dual processing theories, wherein analytic mode and intuitive mode are qualitatively different and should be negatively correlated. Given the positive correlations, our results do not appear to support dual processing models, and instead seem to better align with theorizing from Kruglanski and Thompson’s (1999) unimodal and Reyna and Brainerd’s (1995, 2011) fuzzy-trace theory, both of which predict and empirically demonstrate that analytic and intuitive modes of processing can occur simultaneously (e.g., Kruglanski and Thompson, 1999; Furlan et al., 2016). We also found that, contrary to the hypothesized relationship, motivational threat was positively correlated with intuitive processing (although in the mediation model the coefficient between motivational threat and intuitive mode was not significant). We conceptualized the relationship between motivational threat and analytic mode as positive and the relationship between motivational threat and intuitive mode as negative. However, there is evidence in inoculation research to suggest that inoculation can have heuristic value, producing intuitive thinking: Banas and Miller (2013), for instance, hypothesized and found that “… inoculation can function heuristically as well as through systematic message processing” (p. 189). Thus, consistent with Banas and Miller’s (2013) findings, our correlational results indicate that motivational threat can be associated with both analytic and intuitive (i.e., heuristic) modes of thinking.

The examination of cultural influences revealed that national culture had a limited effect on mechanisms of resistance. We only found that United States participants processed the inoculation message more deliberately using analytic thinking, relative to Finns. This finding is not surprising, given that the *9/11 Truth* conspiracy theory should be personally relevant and resonate more with American participants. When proposing to examine cultural-context variables, we reasoned that using national culture as a predictor would be too broad to fully account for differences across cultural groups (Poortinga and van de Vijver, 1987; Bond and van de Vijver, 2010; van de Vijver and Leung, 2021). However, similar to the effects found for national culture, the results regarding context variables—thinking styles, media literacy, and epistemic trust—likewise revealed limited influences on resistance. For the most part, the significant relationships for cultural-context variables were only related to the thinking modes of message processing. It makes sense that predispositions toward a particular thinking style, being able to understand the source, intent, and meaning behind media messages (i.e., media literacy), and the mode with which messages were processed would have influences on one another. Yet, these were the only significant influences, as cultural-context variables did not affect motivational threat, and only rational thinking style was a significant covariate for resistance.

One possible explanation for why we did not find significant effects of media literacy is that we did not have much variance in this variable because those endorsing the *9/11 Truth* conspiracy theory were excluded from the analyses. As a prebunking (not a debunking) technique (Bessarabova and Banas, 2024; *cf.* Compton, 2020), inoculation should be more effective in defense of pro-attitudinal positions. Our inoculation message would have been counterattitudinal for study participants already endorsing the 9/11 conspiracy theory. Previous research indicates that media literacy is negatively related to conspiratorial beliefs (see Jeong et al.’s, 2012, meta-analysis), which is why, after conspiracy theorists were removed from the study, we only found limited influences of media literacy on the mechanisms of inoculation.

Considering why we did not find cross-cultural differences in epistemic trust in our study, these results are possibly an artifact of the measure we used. We asked participants to indicate how much they trusted religious and educational institutions, as well as interpersonal connections such as friends, family, and neighbors, to do the right thing. This measure focused heavily on interpersonal connections, and it looks like Americans and Finns are more alike rather than different with regard to their trust in interpersonal connections. Regarding conspiracy theories like *9/11 Truth*, mistrust in epistemic authorities like government and governmental agencies that investigated what happened in the aftermath of 9/11 would have been more relevant to examine. Future research should focus on trust in these epistemic authorities rather than interpersonal trust in the context of conspiratorial ideation prevention.

We used a conspiracy prevalent in the United States, and although it was of a lesser relevance for Finnish participants, our results indicated that they found the influence of conspiracy propaganda compelling unless they were in the inoculation condition. The influences of conspiracy theories like these are becoming a global threat. Some conspiratorial propaganda has been disseminated across the world (e.g., the anti-vaccination conspiracy film *Vaxxed*), with long-lasting and resilient deleterious effects. *Loose Change* is an example of a domestic conspiratorial influence that has potential to do damage to audiences abroad by undermining trust in institutions and official narratives. Foreign propaganda can likewise be imported into the United States, posing a significant societal concern with implications for national security (Global Engagement Center, 2020). Understanding what makes foreign conspiracy propaganda persuasive and developing efforts to prevent its influence is an important direction for future research.

Our study used a rather rudimentary method of a counter-propaganda induction. Future investigations should build on a fruitful line of research exploring various different methods of bias mitigation (e.g., Bessarabova et al., 2016; Lee et al., 2016, 2021; Dunbar et al., 2017) and misinformation prevention (e.g., Roozenbeek and van der Linden, 2019; Lees et al., 2023), creating alternative mediated inoculation inductions. Given the benefits of active participation in the inoculation process (Banas and Bessarabova, 2009), exploring the means to facilitate more active engagement in resistance through gamification might be beneficial to misinformation-mitigation efforts. Furthermore, as research on boosters of inoculation has demonstrated (e.g., Parker et al., 2022), repeated engagement with inoculation material is key to sustained resistance. Videogaming provides a natural avenue for repeated exposure (Dunbar et al., 2014) and therefore presents an opportunity to offer inoculation boosters in a nonobvious and engaging manner.

### 4.1 Limitations

As with all research studies, there are limitations to the present experiment. We acknowledge that we used college samples to test our study predictions, and the use of college students in socio-scientific research is often seen as a limitation. However, in cross-cultural research, in particular, the use of college populations can offer important advantages. As van de Vijver and Leung (2021) note, dissimilarity in cross-cultural groups is one of the main problems with cultural research because dissimilarity can result in confounds—demographic differences in age, education level, rural versus urban residence, occupation—invalidating study results. Using student samples makes cultural comparison groups more comparable because college students tend to be more similar in terms of various demographics, and, based on van de Vijver and Leung (2021), minimizing the differences in demographic characteristics can “greatly reduce the number of alternative explanations” (p. 31).

Furthermore, the research comparing the findings from college samples to other sample types suggests that the purported differences in sampling may be exaggerated. For instance, Druckman and Kam (2009) found that on various characteristics—such as ideological differences, partisan beliefs, media use, the role of religion, government, and immigration, views on sexual orientation, perceptions of social trust, engagement in politics and political discussions—students and non-students were more similar than different. A similar conclusion is echoed by Mullinix et al. (2015) who compared political beliefs across student samples, online panels, and probability sampling, revealing “substantively ...similar results” (p. 116). Thus, it appears that employing student samples could be advantageous to cross-cultural research, and the results obtained from such samples could be generalizable to other populations.

Another limitation of the current study is that only a single conspiracy theory and a single inoculation treatment message were examined. Although this provides more experimental control, it also invites critiques regarding generalizability. We encourage future research investigations to examine a variety of inoculation messages as well as other conspiracy theory misinformation with different cultural samples. It is instructive to note that the purpose of data is not to make generalizations but to test them (Mook, 1983). The reality is that every dataset is limited, but although “data are always finite, theory is not constrained in the same way” (Levine, 2011, p. 33).

## 5 Conclusion

The spread of conspiracy theories and misinformation is on the rise, highlighting the need for communication strategies to prevent their proliferation. In this study, an inoculation strategy was successful at inducing resistance to a compelling video from conspiracy propaganda. Our results indicate that inoculation treatment was equally effective for both American and Finnish participants, providing a cross-cultural validation of inoculation effects in combating conspiracy propaganda. These results validate a new measure of motivational threat in a cross-cultural context. We found that motivational threat increased analytic message processing mode but was not related to intuitive processing mode; and regardless of inoculation condition, intuitive processing mode was positively associated with the endorsement of conspiracy propaganda. Although two significant indirect effects have emerged—inoculation ➔ motivational threat ➔ resistance and inoculation ➔ motivational threat ➔ analytic mode of thinking—these results indicate that that the influence of inoculation on conspiracy attitudes is largely a direct effect, with an indirect effect through motivation threat only, as these data did not reveal significant mediation through either analytic or intuitive thinking for the relationship between inoculation and resistance. Finally, examining cultural influences resulted in limited effects of national culture on mechanisms of resistance, demonstrating that United States participants processed the inoculation message more deliberately using analytic thinking, relative to Finns.

## Data availability statement

The raw data supporting the conclusions of this article will be made available by the authors, without undue reservation.

## Ethics statement

The studies involving humans were approved by the University of Oklahoma Institutional Review Board. The studies were conducted in accordance with the local legislation and institutional requirements. The participants provided their written informed consent to participate in this study.

## Author contributions

EB: Conceptualization, Investigation, Formal analysis, Writing – original draft, Writing – review & editing. JAB: Conceptualization, Writing – original draft, Writing – review & editing. HR: Investigation, Writing – review & editing. NT: Formal analysis, Writing – review & editing. VL-a: Resources, Writing – review & editing. KT: Writing – review & editing.

## Footnotes

^1^Given that traditional inoculation is a preemptive strategy (Bessarabova and Banas, 2024; *cf.* Compton, 2020), the data from those who endorsed the 9/11 conspiracy theory at pretest (less than 10% of participants) were removed from the study.

^2^Employing principal axis factoring (PAF) to form indices does not change the study results: All correlations between the indices formed using PCA and PAF were above 0.95 with an average correlation being 0.99.

^3^Given that PROCESS only estimates the indirect effect for the mediation between the independent variable (here, inoculation treatment) and the dependent variable (here, attitudes toward conspiracy film; i.e., resistance), other viable indirect effects were not examined. To estimate the indirect effect for the mediation between inoculation treatment and the analytic mode of processing through motivational threat, Model 4 was used (see Table 4 for details).

^4^We conducted exploratory analyses to ascertain whether national culture interacted with the antecedents in the model. The results of the analyses (Model 92) indicated that there were no significant main effects or interactions between national culture and any antecedent in the model.
